# Lamellarity-Driven Differences in Surface Structural Features of DPPS Lipids: Spectroscopic, Calorimetric and Computational Study

**DOI:** 10.3390/membranes13010083

**Published:** 2023-01-09

**Authors:** Lea Pašalić, Barbara Pem, Danijela Bakarić

**Affiliations:** Division for Organic Chemistry and Biochemistry, Ruđer Bošković Institute, Bijenička 54, 10000 Zagreb, Croatia

**Keywords:** 1,2-dipalmitoyl-*sn*-glycero-3-phospho-L-serine sodium salt (DPPS), multilamellar and large unilamellar vesicles (MLV and LUV), surface curvature fluctuations, interbilayer water, spectroscopic and calorimetric study, MD simulations

## Abstract

Although single-lipid bilayers are usually considered models of eukaryotic plasma membranes, their research drops drastically when it comes to exclusively anionic lipid membranes. Being a major anionic phospholipid in the inner leaflet of eukaryote membranes, phosphatidylserine-constituted lipid membranes were occasionally explored in the form of multilamellar liposomes (MLV), but their inherent instability caused a serious lack of efforts undertaken on large unilamellar liposomes (LUVs) as more realistic model membrane systems. In order to compensate the existing shortcomings, we performed a comprehensive calorimetric, spectroscopic and MD simulation study of time-varying structural features of LUV made from 1,2-dipalmitoyl-*sn*-glycero-3-phospho-L-serine (DPPS), whereas the corresponding MLV were examined as a reference. A substantial uncertainty of UV/Vis data of LUV from which only *T*_m_ was unambiguously determined (53.9 ± 0.8 °C), along with rather high uncertainty on the high-temperature range of DPPS melting profile obtained from DSC (≈50–59 °C), presumably reflect distinguished surface structural features in LUV. The FTIR signatures of glycerol moiety and those originated from carboxyl group serve as a strong support that in LUV, unlike in MLV, highly curved surfaces occur continuously, whereas the details on the attenuation of surface features in MLV were unraveled by molecular dynamics.

## 1. Introduction

The plasma membranes of eukaryotes are lipid- and protein-based bilayer structures that maintain the boundary and regulate the flow of substances between the cellular and extracellular space [1]. Their inherent asymmetry is reflected in the unequal composition of lipids in the opposite leaflets, so the outer leaflet is especially enriched with phosphatidylcholines (PC) and sphingomyelin (SM), while membrane curvature-promoting phosphatidylethanolamine (PE) and phosphatidylserine (PS) [2,3] are almost exclusively found in the inner leaflet [4,5]. Being the most abundant anionic lipid in eukaryotic cells, PS imparts a negative charge in the inner membrane leaflet [6] and exerts a high affinity towards divalent cations, among which Ca^2+^ stands out [7,8,9]. Since the aggregation of PS-containing membranes driven by binding of divalent cations seems to be a prerequisite for the membrane fusion [10,11], the inner leaflet of eukaryotes is more fusogenic than the outer one [12]. Although cell death signaling due to the transition of PS lipids from the inner to the outer membrane leaflet is one of their most important and by far the most famous signatures [13,14], they are also distinguished by some other features, for instance, by their involvement in the aggregation of amyloid formations [15] and the contribution to the enhanced anti-inflammatory effect of certain therapeutics [16].

**Figure 1:**
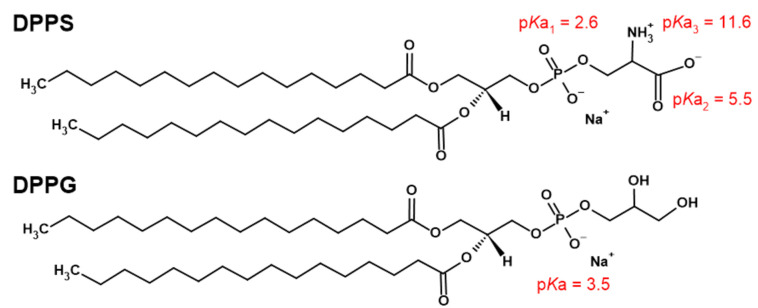
Structural formulas and p*K*_a_ values of particular titrable functional groups of 1,2-dipalmitoyl-*sn*-glycero-3-phospho-L-serine (DPPS) and 1,2-dipalmitoyl-*sn*-glycero-3-phosphoglycerol (DPPG) [18,20].

Since the understanding of the role of individual lipids in eukaryotic plasma membranes is often achieved by analyzing pure lipid membranes, various experimental and computational techniques are used in characterizing the properties of PS-lipid membranes [3,7,8,9,10,17,18,19]. For instance, in fully hydrated membranes constituted from 1,2-dipalmitoyl-*sn*-glycero-3-phospho-L-serine (DPPS), at pH ~ 7, phosphate and carboxylic groups are expected to be deprotonated (p*K*_a1_^app^ ≅ 2.6 [20], p*K*_a2_^app^ ≅ 5.5 [18]) and amino group to be protonated (p*K*_a3_^app^ ≅ 11.55 [18]), resulting in singly negatively charged DPPS lipids which, when suspended in an aqueous solution of *I* (NaCl) = 100 mM, undergo the main phase transition (gel → fluid) at *T*_m_ ≈ 54 °C [18]. Due to the intermolecular interactions involving carboxyl and amino groups [18,21] (Figure 1), the headgroup region of DPPS lipids is more rigid than, for example, those of zwitterionic 1,2-dipalmitoyl-*sn*-glycero-3-phosphatidylcholine (DPPC), which is reflected in both the higher *T*_m_ (*T*_m_ (DPPS) ≈ 54 °C [18] > *T*_m_ (DPPC) ≈ 41 °C [22]) as well as the stiffer packing of DPPS lipid molecules [18,23].

However, as soft and deformable supramolecular structures of high charge density, their response to the exposed stimulus strongly varies with the electromechanical properties of the bilayer [24,25,26], along with the composition and amount of the aqueous medium in which the lipids are suspended [26,27,28]. The instantaneous morphological alterations such as surface curvature fluctuations of DPPS-constituted lipid membrane [29] may drive the local pH change and occasional (de)protonation of carboxylic groups (Figure 1) that primarily depend on the inherent bilayer curvature and associated elastic constants [25,26,29]. Although the rate and the extent of protonation of DPPS lipids in essentially flat multilamellar (MLV) and curved large unilamellar liposomes (LUV) are presumably different, this phenomenon is exceptionally difficult to untangle as purely anionic membranes are highly unstable [25]. Apart from the size and curvature of the system, a crucial fundamental difference in MLV/LUV is the presence/absence of interbilayer forces that maintain the adjacent bilayers in MLV at certain distance. Formed as a result of the balance of repulsive forces (electrostatic and hydration forces) and attractive van der Waals forces [30,31,32], the interbilayer force is dominantly driven by the interplay of the interfacial water layer structural and dynamical features and the surface charge density [33]. In an EPR study of MLV made of dioleoylphosphatidylcholine (DOPC), Ge and Freed demonstrated that ordering of water molecules in the interbilayer region is not only the function of the exchange of water molecules between interfacial region and bulk, but also that this movement is accompanied by the increase in headgroup ordering [34]. As the individual and joint movement of water molecules are constrained by water ordering, this coupling makes interbilayer water more ordered than the bulk one. Moreover, in an XRD and NMR study of MLVs made of DOPC and DOPS, Petrache et al. reported that, regardless of the phase (gel or fluid), less flexible DOPS lipids exert greater perturbation of interbilayer water and greater interlamellar hydration force than in DOPC [35]. Since interbilayer water directly affects surface curvature fluctuations in MLV made of differently charged lipids, its absence in LUV, in which lipids do not experience the suppression of undulations or any limitation in their either individual and collective movement [36], may be of paramount importance in casting the light on the differences in electromechanical properties of MLV and LUV and a subsequent protonation of carboxylic moieties.

In contrast to MLVs, studies of LUVs prepared from DPPS lipids are rather scarce [37,38], especially those related to the characterization of the surface of inherently unstable anionic lipid bilayers. In order to identify the surface structural features, we performed a detailed calorimetric, spectroscopic and a computational study of both LUV and MLV constituted from dominantly DPPS lipids. In order to prevent premature aggregation/lamellarization of LUV, we incorporated small amount of DPPG (*x*(DPPG) = 5%) lipid along with DPPS in both MLV and LUV as it prolongs the lifetime of LUV [39,40,41] (Figure 1). Using DSC and temperature-dependent UV/Vis spectroscopy, we examined thermotropic properties of MLV and LUV and decomposed the melting process of LUV into separated events. The latter were translated into the molecular functional groups-basis by acquiring FTIR spectra of DPPS + 5% DPPG bilayers in gel (L_c/β_ at 35 °C) and fluid (L_α_ at 65 °C) phase (due to the possibility of finding DPPS lipids in both L_c_ and L_β_ phase at 35 °C [21], until we prove the lipids packing pattern in the gel phase, we will write L_c/β_ throughout the text). Along with the signatures of particular moieties of lipid molecules, surface structural features are linked with the hydrogen bond network meshed by polar headgroups and interfacial (LUV) and/or interbilayer (MLV) water layer using MD simulations.

## 2. Experimental

### 2.1. Chemicals and Liposome Preparation

1,2-dipalmitoyl-*sn*-glycero-3-phospho-L-serine sodium salt (DPPS) and 1,2-dipalmitoyl-*sn*-glycero-3-phospho-(1′-rac-glycerol) sodium salt (DPPG) were purchased as white powders from Avanti Polar Lipids (≥99%). The powders were dissolved in chloroform (CHCl_3_; colorless liquid, p.a., Carlo Erba) and their stock solutions (*γ*(DPPS) = 10 mg mL^−1^ and *γ*(DPPG) = 1 mg mL^−1^) were further used in the preparation of multilamellar (MLV) and unilamellar (LUV) DPPS + 5% mol DPPG liposomes, respectively. Briefly, in each flask, 3 mL of DPPS stock solution and 1.480 mL of DPPG stock solution were pipetted in order to obtain the molar fraction of *x*(DPPG) = 5%. CHCl_3_ was removed from the flasks on a rotary evaporator and lipid films were formed as a result, which were completely dried by exposure to Ar stream. In order to obtain MLV suspensions, the films were dissolved in 6 mL of phosphate buffer (PB) of ionic strength (*I* (PB) = 100 mM) and pH ≈ 7.4 (PB was prepared by dissolving the appropriate amounts of NaH_2_PO_4_ and Na_2_HPO_4_ (both supplied from Kemika, p.a.) in Milli-Q^®^ water and its pH was measured using a pH meter) and the flasks with the obtained contents were exposed to at least three cycles of successive vortexing and immersion in, alternately, a hot (~75 °C) and a cold (~4 °C) bath, so that the preparation of the sample lasted about 30 min. The mass concentrations of lipids in MLV prepared in this way were *γ* = 5 mg mL^−1^ for DSC and FTIR and *γ* = 1 mg mL^−1^ for UV/Vis measurements. The LUV constituted from DPPS + 5% DPPG were obtained by extrusion of MLV suspensions using an Avanti^®^ Mini Extruder with holder/heating block through 100 nm size polycarbonate membrane and with the assistance of 10 mm supporting filters (the heating block was heated up to 70 °C to keep the lipids in a fluid state). The syringes were pushed at least 31 times through the apparatus. As LUV composed from DPPS + 5% DPPG lipids are expected to be quite unstable [25], the prepared MLV suspension was extruded before each measurement set (DSC, UV/Vis and FTIR) and the size distribution of obtained LUVs was determined using dynamic light scattering (DLS) prior to the corresponding (thermoanalytical and spectroscopic) measurement. Additionally, imaging on a confocal microscope was done to detect eventual traces of MLV in LUV suspension.

### 2.2. Dynamic Light Scattering (DLS) and Confocal Microscopy: Measurements and Data Analysis

The size distribution of liposomes was established with dynamic light scattering using a photon correlation spectrophotometer equipped with a 532 nm (green) laser (Zetasizer Nano ZS, Malvern Instruments, Worcestershire, UK). The average hydrodynamic diameter (*d*_h_) was specified as the value at peak maximum of the volume distribution. The reported results correspond to the average of six measurements at 25 °C. The data processing was done with the Zetasizer software 7.13 (Malvern Instruments). The average hydrodynamic diameters values of MLV constituted from DPPS + 5% DPPG (at *γ* = 0.05 mg mL^−1^) were in the range 300 nm ≤ *d*_h_ ≤ 500 nm. The sizes of LUV of DPPS + 5% DPPG were in the range 110 nm ≤ *d*_h_ ≤ 115 nm for (DSC and) UV/Vis measurement, 125 nm ≤ *d*_h_ ≤ 137 nm for DSC and confocal microscopic imaging (at *γ* = 5 mg mL^−1^) and 107 nm ≤ *d*_h_ ≤ 120 for FTIR ATR measurement and DSC (Figure S1 in Supplementary Materials). The white light laser source of the Leica TCS SP8 confocal microscope (Leica Microsystems, Wetzlar, Germany) was used for imaging of MLV and LUV suspensions (*γ*(DPPS + 5% DPPG) = 5 mg mL^−1^). Using a 63× (N.A. = 1.4) oil-immersion objective, the images were collected in reflective and transmissive modes (Supplementary Materials, Figure S2).

### 2.3. Differential Scanning Calorimetry (DSC): Data Acquisition and Curve Analysis

The calorimetric experiments were carried out in a microcalorimeter Nano-DSC, TA Instruments (New Castle, DE, USA). Suspensions of DPPS + 5% DPPG (*γ*(DPPS + 5% DPPG) = 5 mg mL^−1^) were held for 10 min in a degassing station before starting the measurement. Both MLV and LUV suspensions were recorded at a scan rate of 1 °C min^−1^ at least two times in two repeated heating-cooling cycles in a temperature range of 40–65 °C. Additionally, MLV suspension was examined in a temperature range 25–65 °C in order to detect eventual existence of L_c_ phase, whereas LUV suspension was additionally measured in the range 35–65 °C (see Figures S3 and S4 in Supplementary Materials). PB (a reference scan) was examined once in the temperature range 10–90 °C. Data analysis was performed using the TA Instruments Nano Analyze software package as follows: (i) the DSC curve of reference solution (PB) was subtracted from the raw DSC curve of the explored suspensions; (ii) the baseline correction of DSC curves in the temperature range of interest was made. The phase transition temperature was determined from both onset (_o_) and maximum (_m_) of the DSC curve (*T*_m, o/m_) [42] from the first heating run in order to eliminate possible impact of heating and cooling on inherently unstable anionic lipid bilayers [25] and on the protonation of negatively charged DPPS lipids (both heating and cooling runs of MLV and LUV are presented in Figures S5 and S6). A series of trial DSC measurements (along with accompanied DLS) were conducted in order to estimate the time period during which LUVs do not aggregate/lamellarize (which results with MLV formation) and it was estimated that during 6 hours, the amount of time it takes to perform a DSC measurement (including sample degassing and pressure/current stabilization), one can obtain relatively reproducible results for LUV (more details on are provided in Supplementary Materials, Figure S4). In this light, the best solution for obtaining as reproducible as possible DSC curves was to extrude a certain volume of the original MLV suspension immediately before the DSC measurement. Despite the lack of representative reproducibility, such as that observed in the DSC curves of MLV suspensions, this turned out to be the best that could be achieved (more details on both heating and cooling runs in DSC measurements of MLV and LUV are shown in Supplementary Materials, Figures S5 and S6).

### 2.4. UV/Vis Spectroscopy: Data Acquisition and Spectral Analysis

UV/Vis spectra of MLV and LUV of DPPS + 5% DPPG (*γ* = 1 mg mL^−1^) were measured on the UV/Vis spectrophotometer Thermo Scientific Nanodrop 2000 (Thermo Fisher Scientific, Waltham, MA, USA) in the spectral range of 200–500 nm. The spectra of MLV and LUV suspensions were recorded at least three times (in three different cuvettes) in the temperature ranges 30–65 °C and 40–65 °C, respectively. The spectra of PB were collected once in the respective temperature ranges.

After acquisition, the spectra were smoothed using Savitzky-Golay (10 points, polynomial of a third degree) [43] and the spectral range 250–300 nm was subjected to multivariate curve analysis (MCA) examined using publicly available MATLAB code [44]. The aforementioned approach enables the projection of temperature-dependent UV/Vis spectra onto a certain number of components, which contain all the information contained in the original data or spectra. Since in this case the total variance in the spectral set (**D**) can be described by one component, solving the equation

**D** = **CS^T^** + **E** (1)

where (**C**) is the concentration, and (**S**) is the spectral profile of the (first) principal component, and (**E**) is the residual matrix, is possible. More details on this multivariate approach can be found in, for instance, [44,45,46,47,48].

A common feature of the temperature-dependent concentration profiles of DPPS-constituted MLV and LUV is their sigmoid character, and the crucial difference between them is the number of sigmoidal transitions in the investigated temperature range; statistically, the obtained curve for MLV curve is the best to fit on a double Boltzmann profile (*R*^2^ = 0.998 and *χ*^2^ = 34.1 for a single Boltzmann and *R*^2^ = 0.999 and *χ*^2^ = 0.666 for a double Boltzmann), whereas the one for LUV only a single Boltzmann fit gave meaningful values (*R*^2^ = 0.882 and *χ*^2^ = 9.5 for a single Boltzmann).

### 2.5. FTIR ATR Spectroscopy: Data Acquisition and Spectral Analysis

FTIR ATR spectra of MLV, LUV and PB were collected on Invenio-S Bruker spectrometer, equipped with the photovoltaic LN-MCT detector, using a BioATR II unit. The latter is circular with radius of 2 mm and is based on dual crystal technology, where the upper ATR crystal is made of silicon and the lower ATR crystal is made of ZnSe. The inside of the ATR unit was continuously purged with N_2_ gas connected with external supply and temperature-controlled using circulating water bath of Huber Ministat 125 temperature controller. The suspensions (MLV and LUV of mass concentration *γ*(DPPS + 5% DPPG) = 5 mg mL^−1^) and PB were pipetted directly on the ATR crystal unit in a volume of 30 μL and their spectra were acquired against air as a background. Using OPUS 8.5 SPI (20200710) software, all spectra were collected at nominal resolution of 2 cm^−1^ and 256 scans at two different temperatures, namely 35 °C (L_β/c_) and 65 °C (L_α_). Each suspension was measured three times, whereas PB solution was measured once.

The FTIR spectra were examined in spectral ranges that display vibrational signatures of the most relevant functional groups: (i) 2980–2820 cm^−1^ (ν_(a)s_CH_2_), (ii) 1780–1530 cm^−1^ (νC=O, ν_as_COO^−^), (iii) 1505–1395 cm^−1^ (γCH_2_, ν_s_COO^−^, δCOH) and (iv) 1255–1190 cm^−1^ (ν_as_PO_2_^−^, ν_a(s)_C−O). Before analysis the spectral parts were smoothed using the Savitzky-Golay approach (i) 10 points-, (ii) 50 points-, (iii) 30 points- and (iv) 30 points-cubic polynomial) and baseline corrected. The positions and shapes of the bands of interests were determined and discussed for DPPS MLV/LUV found in the gel (L_β/c_: 35 °C) and in the fluid (L_α_: 65 °C) phase.

## 3. Molecular Dynamics Simulations

Classical molecular dynamics (MD) was employed to model DPPS membranes with 5% DPPG, in gel and fluid phase. The membranes, consisting of 122 DPPS molecules and 6 DPPG molecules (distributed evenly in both leaflets), were constructed using the CHARMM-GUI Membrane Builder module [49], and the negative charge was neutralized by the addition of 128 Na^+^. In order to simulate the membrane interactions in LUV vs. MLV, two different setups were used: system 1 was solvated with 6400 water molecules, whereas system 2 was solvated with 2781 water molecules. Periodic boundary conditions were instituted in both cases, however in system 1 the length of the box *z*-axis was equilibrated to 9.4 nm (fluid) or 11.2 nm (gel), so the periodic images were separated by 3.8–5.2 nm of bulk water. Since the images were separated by more than 2 times the non-bonded interactions cutoff (1.2 nm), the membrane was unable to interact with its periodic images and thus simulated a unilamellar system (UL). System 2’s *z*-axis lengths after equilibration were 6.5 nm (fluid) and 7.5 nm (gel), meaning the periodic images were separated by only 1.0–1.8 nm of a water layer, and essentially formed an infinite multilamellar structure (ML). The amount of water in multilamellar simulations was chosen to fit the required box size to correspond to literature reports of DPPS bilayer repeat distance (6.3–6.8 nm) in fluid phase MLVs [50,51]. Gel phase membranes were simulated at 35 °C, and the fluid phase at 65 °C, corresponding to the temperatures used for FTIR measurements.

All simulations were run using the GROMACS 2020.0 software [52], CHARMM36m force field [53] and the TIP3P water model [54]. Following the energy minimization, heating was conducted for 200 ps in the NVT ensemble with the V-rescale algorithm, and the simulations were run for 300 ns in the NpT ensemble (Nosé-Hoover thermostat [55] with the time constant of 1 ps; Parrinello-Rahman barostat [56] with target pressure of 1 × 10^5^ Pa, semi-isotropic pressure coupling and the time constant of 5 ps). The first 150 ns of production were discarded as equilibration time, and only the last 150 ns were used for analysis. As mentioned, the cutoff for short range Coulomb interactions and van der Waals interactions was 1.2 nm with the switching function for the latter turned on after 1 nm. The particle mesh Ewald (PME) procedure [57] was utilized for long-range Coulomb interactions. The time step for all simulations was 2 fs, and all bonds involving hydrogen atoms were constrained using LINCS. GROMACS modules and Visual Molecular Dynamics (VMD) [58] were used for data analysis.

## 4. Results

### 4.1. Thermotropic Properties of DPPS Lipids: UV/Vis and DSC Data

**Figure 2:**
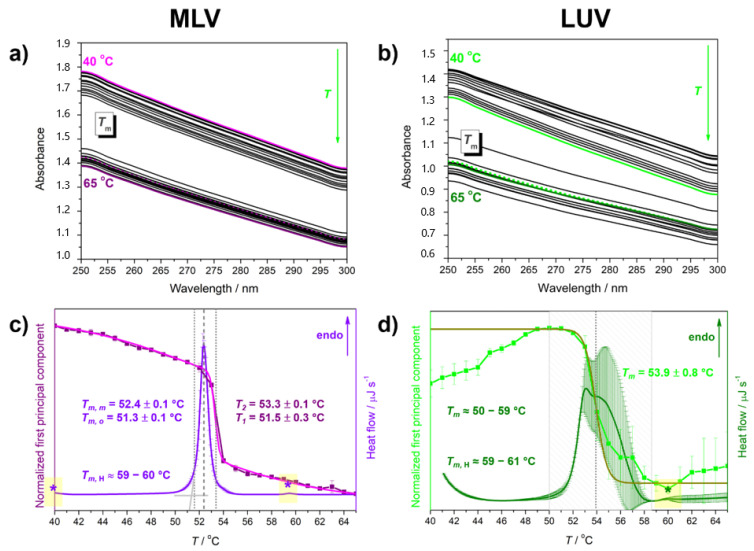
Temperature-dependent UV/Vis spectra of DPPS in the following forms: (**a**) MLV (40 °C: magenta solid curve, 65 °C: purple solid curve, spectral profile of the principal component: magenta dotted curve) and (**b**) LUV (40 °C: green dotted curve, 65 °C: olive solid curve, spectral profile of the principal component: green dotted curve). Concentration profiles of the (first) principal components that project temperature-dependent UV/Vis spectra and DSC curves of DPPS: (**c**) MLV (DSC: violet curve; UV/Vis: purple curve for spectral projection and magenta curve for double sigmoidal fit) and (**d**) LUV (DSC: olive curve; UV/Vis green curve for spectral projection and dark yellow curve for single sigmoidal fit). Phase transition temperatures determined from DSC (intersected lines for _o_ and dashed lines for _m_) and UV/Vis experiments (dotted lines) are highlighted with corresponding colors on graphs. * and yellow rectangles refer to the L_c_ → L_β_ (in MLV only) and *T*_m,H_ of DPPS (in both MLV and LUV).

In the characterization of the thermotropic properties of DPPS lipids in MLV, there are no excessive surprises or deviations from DSC values known in the literature [59,60]; MLV suspensions become more and more transparent due to heating with a pronounced discontinuity in the absorbance decrease at the L_β/c_ → L_α_ phase transition [61] (Figure 2a). By fitting the temperature-dependent concentration profile of the (first) principal component curve on the double Boltzmann function (Figure 2c), we obtained the values of inflection points at *T*_m,1_ = 51.5 ± 0.3 °C and *T*_m,2_ = 53.3 ± 0.1 °C (Table 1). The first value coincides with *T*_m,o_ (51.3 ± 0.1 °C), whereas the latter one is for about 1 °C higher than *T*_m,m_ (52.4 ± 0.1 °C) determined from the DSC curve (Figure 2c). The DSC curve of MLV of DPPS lipids displays two additional weak endothermal events (both labelled with * and a yellow rectangle in Figure 2c): (i) the one with the maximum at about 37 °C originated from the L_c_ → L_β_ [21] (here we displayed only the range 40–65 °C, whereas in Supplementary Materials in Figure S3, we displayed a broader temperature range) and another one almost unnoticed at about 60 °C originated from the melting of a small fraction of protonated DPPS lipids at pH = 7.4 (*T*_m,H_ ≈ 60 °C) [18,62,63] (Table 1).

In contrast to the temperature-dependent UV/Vis spectra of MLVs, there is no gradual decrease in absorbance with increasing temperature for LUVs (please see and compare the spectra of MLV and LUV at marginal temperatures in Figure 2a,b); on the contrary, except for a sharp decrease in absorbance at the temperature of the L_β/c_ → L_α_ phase transition (*T*_m_), the absorbance before and after *T*_m_ seems to change in a manner different than MLV, but still maintains the reproducibility (Figure 2b). Such a trend is also reflected in the appearance of the concentration profile of the component onto which the UV/Vis spectra were projected, which shows one point of inflection, i.e., one significantly cooperative event (L_β/c_ → L_α_ phase transition), with uncertainty larger than in MLV (Figure 2d). Regarding the DSC curve of (Figure 2d), the first thing that catches the eye is the great uncertainty in melting profile of LUV, but exclusively at the high-temperature wing of the curve. According to the obtained averaged DSC curve, the melting of deprotonated DPPS lipids in LUVs occurs in an extremely wide temperature interval (50–59 °C, Table 1) [64] (for more details see Supplementary Materials, Figure S5), which is expected for systems in which the lipid domains subject to cooperative motion are significantly smaller [65,66], whereas the melting of protonated DPPS lipids (COOH) occurs in a temperature interval slightly wider than that of MLV (*T*_m, H_ ≈ 59–61 °C in LUV contrast to ≈60 °C in MLV, Table 1)).

### 4.2. Molecular Properties of DPPS Lipids in Gel and Fluid Phase: FTIR Data

**Figure 3:**
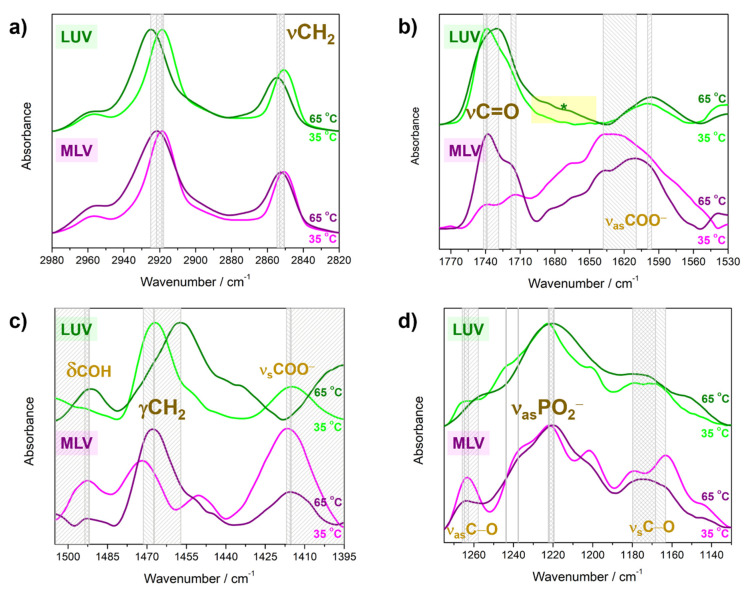
Normalized FTIR spectra of DPPS in the forms of MLV (35 °C: magenta, 65 °C: purple) and LUV (35 °C: green, 65 °C: olive) in the following spectral ranges: (**a**) 2980–2820 cm^−1^ (ν_(a)s_CH_2_), (**b**) 1780–1530 cm^−1^ (νC=O, ν_as_COO^−^), (**c**) 1505–1395 cm^−1^ (γCH_2_, ν_s_COO^−^, δCOH) and (**d**) 1255–1190 cm^−1^ (ν_as_PO_2_^−^, ν_a(s)_C−O). * Temperature-dependent displacement of the bands are highlighted with rectangular filled pattern.

In the reconstruction of the most relevant molecular-level events, we paid special attention to the analysis of spectral ranges that reflect conformational changes of hydrocarbon chains upon L_β/c_ → L_α_ phase transition (ν_s_CH_2_ and ν_as_CH_2_ in Figure 3a), packing pattern of lipid molecules (γCH_2_ Figure 3c) and the hydration-related features in the interfacial region (νC=O and ν_as_COO^−^ in Figure 3b, ν_s_COO^−^ in Figure 3c and ν_s_C−O, ν_as_C−O and ν_as_PO_2_^−^ in Figure 3d).

FTIR spectra of MLV/LUV acquired at 35 °C and 65 °C in 2980–2820 cm^−1^ show that the bands originated from antisymmetric (ν_as_CH_2_) and symmetric (ν_s_CH_2_) stretching of methylene groups that, in accordance with expectations, show a high-frequency shift due to the phase change: in the L_β/c_ phase (35 °C), their maxima appear at 2918 cm^−1^/2919 cm^−1^ (ν_as_CH_2_) and 2851 cm^−1^/2850 cm^−1^ (ν_s_CH_2_), whereas in the L_α_ phase (65 °C), they appear at 2922 cm^−1^/2925 cm^−1^ (ν_as_CH_2_) and 2853 cm^−1^/2855 cm^−1^ (ν_s_CH_2_) (Figure 3a).

The region that encompasses the signatures of glycerol backbone and carboxyl moiety of DPPS displays some significant differences in MLV/LUV (Figure 3b). In particular, FTIR spectrum of MLV in L_β/c_ phase (35 °C) apparently displays two envelopes: one with two almost equal maxima at 1742 cm^−1^ and 1716 cm^−1^ attributed to the stretching of carbonyl groups (νC=O) excluded from and included in a hydrogen bond network meshed by interfacial water layer, respectively, and another very broad, intense und poorly structured envelope with the maximum at 1638 cm^−1^ originated from the antisymmetric stretching of COO^−^ moiety (ν_as_COO^−^). Upon transition to the L_α_ phase (65 °C), the first envelope gets slightly narrower and the corresponding maxima, of which the high-frequency one has a stronger intensity than the low-frequency one [21], are found at 1738 cm^−1^ and 1718 cm^−1^, whereas the broad ν_as_COO^−^ band with maximum displaced to 1610 cm^−1^ becomes of comparable intensity with overall the νC=O signature (both non- and a hydrogen-bonded (HB) one). The analogous signatures of LUV differ in terms of relative intensities of overall νC=O and ν_as_COO^−^ bands, the low-frequency wing associated with overall νC=O signature and in the position and magnitude of phase transition-induced displacement of ν_as_COO^−^ band. First, the change in the phase (temperature) results in the displacement of overall νC=O signature from 1741 cm^−1^ (L_β/c_ phase at 35 °C) to 1729 cm^−1^ (L_α_ phase at 65 °C) and in a simultaneous change in the band shape associated with the increase of subpopulation of carbonyl groups involved in a hydrogen bond network with surrounding water molecules [67]; second, the low-frequency wing of this band increases as the temperature rises to the extent that the envelope does not even reach minimum which is observed for MLV (at about 1695 cm^−1^); third, the band originated from ν_as_COO^−^ is of significantly lower intensity and undergoes much smaller low-frequency shift as the phase changes: from 1599 cm^−1^ (L_β/c_ phase at 35 °C) to 1597 cm^−1^ (L_α_ phase at 65 °C). Moreover, the residual signal of interlamellar water in MLV (δHOH; absorbs at about 1640 cm^−1^) may also participate in shaping the discussed envelope of MLV, which is absent in the corresponding spectra of LUV.

The spectral region 1505–1395 cm^−1^ is comprised from the signatures of methylene (γCH_2_) and carboxyl groups (ν_s_COO^−^ and δCOH) (Figure 3c). The position of γCH_2_ signature at 35 °C suggest that in MLV (1472 cm^−1^) and LUV (1467 cm^−1^), DPPS molecules are differently packed: in MLV, the lipids experience tighter lateral interaction and are arranged in a L_c_ phase, unlike in LUV, in which lipids form a L_β_ phase. Their position at 65 °C, which is 1468 cm^−1^ for MLV and 1457 cm^−1^ for LUV, not only implies the increased mobility of hydrocarbon chains, but also that lateral interactions between lipids in LUV are probably affected by some phenomenon that either does not exist in MLV or is considerably weaker compared to that in LUV. The band originated from δCOH slightly shifts from 1492 cm^−1^ (35 °C) to 1494 cm^−1^ (65 °C) in MLV, opposite to the analogous band in LUV that displaces from 1505 cm^−1^ (35 °C, the spectrum is cut-off on the band maximum) to 1492 cm^−1^ (65 °C). Ultimately, the second half of the COO^−^ (moiety signature (ν_s_COO^−^) in MLV barely reports some change in the position −1417 cm^−1^ at 35 °C to 1416 cm^−1^ at 65 °C—which is in contrast to the analogous signature in LUV—and 1415 cm^−1^ at 35 °C to 1396 cm^−1^ at 65 °C (the spectrum is cut-off near the band maximum).

The spectral region in which phosphate group signatures, along with some signatures of glycerol backbone, are found are displayed in Figure 3d. The presence of a more structured envelope in MLV (i.e., sharper maxima) generally suggests a stronger progression of CH_2_ wagging bands (ωCH_2_) that also appear in this spectral range [68]. Regarding the maxima of ν_as_PO_2_^−^ bands associated with the stretching of their non-HB and HB subpopulations (hydrogen bond established most likely with interfacial water molecules), their positions in MLV remain relatively unchanged upon phase transition: non-HB ν_as_PO_2_^−^ appears at 1236 cm^−1^ at both 35 °C and 65 °C, whereas HB one barely displaces from 1222 cm^−1^ (35 °C) to 1221 cm^−1^ (65 °C). In LUV, the analogous signatures merge upon phase transition: from two maxima at 1244 cm^−1^ and 1223 cm^−1^ (35 °C) to one broad envelope with maximum at 1220 cm^−1^ (65 °C). In diagnostic purposes, the commonly not particularly useful signature of ν_(a)s_C−O reflects some interesting differences this time; displacement of ν_as_C−O from 1263 cm^−1^ (35 °C) to 1265 cm^−1^ (65 °C) in MLV is accompanied with a broad feature (ν_s_C−O) that spans the region 1190–1150 cm^−1^ at 35 °C and becomes structureless at 65 °C. In LUV, the ν_as_C−O band maximum shows a greater shift than in MLV and in opposite direction, from 1265 cm^−1^ (35 °C) to 1256 cm^−1^ (65 °C). As for the ν_s_C−O band, it gets broader as lipids undergo phase transition and spans a greater spectral range than the corresponding feature of MLV, from 1190 cm^−1^ at 35 °C to 1130 cm^−1^ at 35 °C.

### 4.3. Molecular Properties of DPPS Lipids in Gel and Fluid Phase: Molecular Dynamics Data

The purposes of MD simulations are the following: (1) to investigate the structural parameters of DPPS + 5% DPPG membranes and the possible differences in unilamellar (UL) vs. multilamellar (ML) setups; and (2) characterize the hydration and effects of water on both interfaces. The visualization of UL and ML setups and the final appearance of the membranes after 150 ns of production are shown in Figure S7 in Supplementary Materials.

The structural parameters of membranes include area per lipid (APL), membrane thickness, deuterium order parameters (S_CD_) and mass density profiles. APL was calculated as the product of box *x* and *y* dimensions divided by the number of lipids in one leaflet, and the averages of 1500 frames of simulation are shown in Table 2. The obtained results correspond well to previous computational [51,69] and experimental [18,70] reports, which obtained APL of 0.44–0.48 nm^2^ (gel) and 0.54 nm^2^ (fluid). Membrane thickness was determined as the average distance between P-atoms of opposing leaflets, obtained from the positions of peak maxima of density profiles, and the error was estimated from the difference between symmetrized and unsymmetrized density calculations. Calculated membrane thickness (Table 2) is inversely correlated with APL and matches the literature value of 4 nm (gel) [51]. Deuterium order parameters are the measure of the disorder of acyl chains and are therefore the indicators of membrane rigidity. At 35 °C, the -S_CD_ values of both chains plateau around 0.25–0.30, pointing to a high degree of structure throughout the full length of the membrane (Figure S8). At 65 °C, the -S_CD_ values are lower in general, with order particularly diminished towards the chain ends (0.10–0.15). The values shown here were higher compared to literature reports of experimental and computational -S_CD_ determination for DPPS (0.1–0.2). However those values were obtained at higher temperatures (77 °C) where more disordering is expected [71]. Lower APL, higher thickness and higher -S_CD_ at 35 °C are indicators of tight packing and high rigidity, which confirms that the membrane is in gel phase, contrasting the high APL, low thickness and low -S_CD_ of the membrane at 65 °C (fluid phase). However, there appears to be almost no difference between UL and ML setups at 35 °C. At 65 °C the ML membrane is thicker and more ordered, but APL values do not differ significantly.

Mass density profiles show the average distribution of atoms and structures along the membrane normal (Figure S9). DPPS headgroups delineate the outer borders of the membrane, and DPPG headgroups are more withdrawn towards the center. The profiles show significant accumulation of water at the interface, the headgroups in both UL and ML systems are well hydrated, and some molecules penetrate further towards the glycerol moiety and acyl chains. Cation binding to the negatively charged membrane surface is also demonstrated by the almost complete overlap of Na^+^ peak density with the headgroups.

To evaluate the membrane hydration, radial distribution functions (RDFs) were calculated for water oxygen (O_WAT_) with lipid molecules as reference. RDFs demonstrate the probability of finding the O_WAT_ atom at a certain distance *r* of the reference group. The RDF profiles (Figure S10) were similar for all systems: a small first peak with maximum at 0.18 nm likely corresponds to lipid-water hydrogen bond formation where the O_WAT_ is the acceptor; the main well-structured peak at 0.27 nm (from H-bonds where O_WAT_ is the donor) points to the existence of a defined first hydration shell; and further broader peaks of secondary or tertiary hydration shells exist where the structuration is not as pronounced. The minimum in the RDF profiles at *r* = 0.3 nm was considered to be the edge of the first hydration shell and was used in further analyses of solvent orientation and bonding.

**Figure 4:**
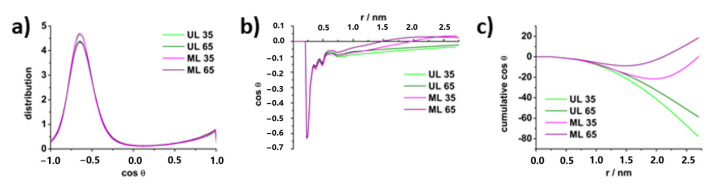
(**a**) angular distribution functions for water molecules in the first hydration shell of DPPS and DPPG lipids; (**b**) tilt of water molecules as a function of distance from the membrane; (**c**) the sum over all solvent molecules of cosθ as a function of distance from the membrane.

The orientation and arrangement of water at the membrane interface may be determined by calculating water angular distribution, expressed as cosine of the angle θ formed by the vector pointing from the lipid headgroup to the water oxygen and the vector from water oxygen to the midpoint between water hydrogens. Random orientation of waters would result in equal probability of all values of cosθ [72,73]. As seen in Figure 4a, the angular distribution function of waters in the first hydration shell yielded the maximum at −0.65, or 130°, meaning the preferred water orientation is with O_WAT_ and one of the H_WAT_ pointing towards the headgroups. Both O_WAT_ and H_WAT_ may participate in hydrogen bonding with lipids, resulting in such positioning, as was also seen in our previous study [39]. Though the angular distribution is identical for all simulations, meaning both UL and ML systems have the same organization of the first hydration shell, the distribution of cosθ vs. distance from the lipid shows the changes in water orientation between the membrane interface and bulk region (Figure 4b). Within approx. 0.7 nm from the membrane, there is no difference between UL and ML—the same structured first hydration shell characterized by cosθ −0.65 and other minima at further distance indicating some structuration in the secondary and tertiary shells. In UL systems, the cosθ remains negative for all values of *r* with the tendency towards 0, meaning the cosθ values began averaging out as one moves from the membrane towards the bulk. However, in ML systems, the values of cosθ become positive at around 1.5–1.9 nm, since the waters feel the pull of the opposite membrane. The difference is more pronounced in the graph of cumulative cosθ vs. the distance (Figure 4c).

In addition, the water order was evaluated by calculating the angle φ between water dipoles and the box *z*-axis as a function of box height (Figure S11). The values of cosφ start at 0 at the edge of the box, reach maximum positive values in the area of headgroups and then drop to maximum negative values approaching the acyl chains (the changing point being in the area of the phosphate group). This means that the water dipoles closer to the membrane center point towards the acyl chains, and those closer to the surface point away, similarly to previous observations [74,75,76]. While this is similar for all systems, the UL and ML profiles again differ in water orientation further away from the interface. In UL, there is a gradual decline in cosφ towards 0 (random) approaching the edge of the box; in ML, the water has clear order throughout and simply flips from positive to negative cosine values when the periodic boundary is crossed. The same inflection point in systems with small interbilayer distances was shown in Pimthon et al. [77].

As previously mentioned, DPPS and DPPG may form hydrogen bonds both with water and with each other. Lipid-lipid and water-water HB were quantified and normalized for the number of molecules per each system (Table 3). The HB of water with whole lipids or individual groups within lipids (phosphate PO_2_, carbonyl C=O, carboxyl COO and glycerol moieties) were not normalized. The criteria for recognizing a HB were having a donor-acceptor distance of <0.3 nm and a hydrogen-donor-acceptor angle of <30°. The average number of lipid-lipid bonds is 1.4–1.7 per lipid, which is higher than 0.8 reported by Pandit et al. [51]; however, this is likely due to differences in setup and criteria. Lipid-lipid and water-water HB are decreased in the fluid phase compared to the gel, while lipid-water HB increases with temperature. The average number of water-water HB in ML is reduced compared to UL. Furthermore, the average number of HB of water with phosphate, carbonyl, carboxyl and glycerol moieties, or lipids overall, are marginally higher in UL compared to ML, though the effect is smaller than standard deviation. In addition, the average number of Na^+^ ions in contact with DPPS COO^−^ was counted and no difference was found between UL and ML systems (Table S1).

Along with structuring and bonding of water, we evaluated the water diffusion coefficient *D* (Table 4). Diffusion coefficients from MD simulations are based on the mean square displacement of an atom or molecule from its initial position in the duration of the simulation, and are calculated using the Einstein’s equation [78,79]:

(2) $$D=\underset{t\;\to \;\infty }{\lim}\sqrt{\frac{\sqrt{{\left(r\left(t\right)-r\left(0\right)\right)}^{2}}}{6t}}$$

where *r(t)* is the position of a particle at time *t* and *r*(0) is the initial position of a particle. The calculated values of *D* are presented in Table 4. The experimentally determined *D* of water at 50 °C is 4.46 × 10^−5^ cm^2^ s^−1^ [80] is in good agreement with the calculated value in UL system at 65 °C, leading to the conclusion that the membrane has only a small effect on water mobility in UL systems. In ML systems, on the other hand, *D* values are 1.7–1.8 times smaller, meaning water molecules have lower mobility in the interbilayer space. In addition, the lateral diffusion coefficient *D_xy_* was calculated for membrane lipids from mean square displacements in the *xy* plane of lipid P-atoms (Table 4). As expected, lipids show a much higher mobility in fluid phase than in gel. In gel phase, the movement of lipids is significantly reduced in ML systems compared to UL systems; however, the discrepancy is minimal in fluid phase.

## 5. Discussion

The presented experimental and computational results of MLVs and LUVs made of anionic DPPS (+5% DPPG) lipids show significant differences in both thermotropic and molecular properties. Since the calorimetrically available thermodynamic properties are a function of not only of the composition and phase of the system, but also of its size [39,81,82,83], the complexity and especially the poor reproducibility of the melting profile on the high-temperature side of dominantly anionic LUVs, in contrast to MLVs, suggest possible presence/absence of curvature-related and time-varying structural changes in LUV/MLV. This hypothesis is additionally supported by the turbidity-related results obtained by multivariate analysis of UV/Vis spectra; with the exception of a sudden change in absorbance at *T*_m_, the usual trend of decreasing absorbance with temperature was not observed in the LUV suspension, which might be attributed to significant inherent fluctuations in the curvature of LUV [39,82,83].

Although the temperatures of the main phase transition obtained calorimetrically (DSC) and spectroscopically (UV/Vis) generally show a satisfactory agreement (Table 1), two exceptions were observed: (i) Unlike DSC measurements in which melting is shown as a single maximum, the concentration profile of the projection of the UV/Vis spectra of MLV has two inflection points separated by about 2 °C (see Table 1). By analogy with DPPC MLV and multilamellar aggregates of DPPE, it is possible that the fluctuations in surface undulations reach their maximum (*T*_1_ = 51.5 ± 0.3 °C) only slightly below melting (*T*_2_ = 53.3 ± 0.1 °C). Effectively, in DPPS lipids, unlike DPPC [39,61,84] and DPPE [84], reaching the maximum of surface curvature fluctuations and the melting itself are almost coupled events. This interpretation is supported by the finding that the repulsions between charged species (DPPS) make anionic (DPPS + 5% DPPG) lipid membranes generally stiffer than the zwitterionic (DPPC and DPPE) ones [35] and suppress the membrane undulations [85]. Due to unfortunately large uncertainty associated with the UV/Vis response of LUV that can easily camouflage an inherently weak response of surface curvature fluctuations [84], only one inflection point can be detected with certainty; (ii) The presence of a certain amount of protonated DPPS lipids (i.e., protonated carboxyl group) was confirmed by DSC, but not by UV/Vis measurements. Although their response is barely detectable in MLV (*T*_m,H_ ≈ 60 °C, Figure 2c), it is reproducible enough to be confidently identified and confirmed by agreement with literature [63].

In addition to the mentioned exceptions, it is necessary to point out the principal difference between MLVs and LUVs, which represents the leitmotif of this manuscript. Contrary to MLV, the intrinsically wide melting range of deprotonated DPPS lipids in LUVs is further broadened due to pronounced uncertainty on the right-hand side of the abscissa as well as along the ordinate (Figure 2d and Figure S6), even to the extent that it extends to the barely perceptible melting of protonated DPPS lipids (59–61 °C, Figure 2d). The relatively unequivocal detection of two maxima (at ~53 °C and ~55 °C) presumably originated from two relatively separate background processes, suggesting that the melting of DPPS (with 5% DPPG) results from the change in the mean curvature (presumably at around 53 °C), which is reflected in the associated elastic constants [26], and from the breaking of van der Waals interactions (presumably at around 55 °C) [39,83]. Since the elastic constants of pure anionic lipid bilayers are modulated by an electrostatically-induced increase in curvature instability [25], it does not seem impossible that surface curvature fluctuations in LUVs of DPPS introduce high uncertainty/variability into the melting once when van der Waals forces between hydrophobic chains are weakened (in zwitterionic lipid bilayers this is usually seen for lipids in L_β_ phase [84]). Surface curvature fluctuations could be expressed either from heat capacity ($c_{p}$)

(3) $$c_{p}=\frac{d\langle H\rangle }{dT}=\frac{\langle H^{2}\rangle -{\langle H\rangle }^{2}}{RT^{2}}$$

or using isothermal area compressibility ($\kappa_{A}^{T}$)

(4) $$\kappa_{A}^{T}=\frac{\langle A^{2}\rangle -{\langle A\rangle }^{2}}{\langle A\rangle RT}$$

where values in numerators in (2) and (3) refers to the fluctuations in enthalpy (*H* in (2)) and area (*A* in (3)) [26].

$\kappa_{A}^{T}$ is essentially the measure of the degree of change in membrane area in response to application of lateral force, and is related to the measure of energy required to for a membrane to bend, the bending modulus ($K_{A}$)

(5) $$K_{A}=\frac{d^{2}}{16\;\kappa_{A}^{T}}$$

where *d* is membrane thickness [26]. High $\kappa_{A}^{T}$ and low $K_{A}$ are required for pronounced membrane curvature. It has been observed both experimentally and computationally that PS lipids have low compressibility and high bending modulus compared to zwitterionic lipids such as PC [35,86,87], but the deformability of DPPS (+5% DPPG) has not been discussed in terms of LUV and MLV. Since $\kappa_{A}^{T}$ is inversely proportional to membrane area and proportional to area fluctuations, and $K_{A}$ is proportional to membrane thickness, the MD simulations were employed to estimate APL (and its standard deviation) and thickness (P–P distance). However, while the experimental results strongly point to higher deformability of LUV compared to MLV, the MD simulations are less conclusive. As seen from Table 2, there is only a small indication that multilamellar membranes are thicker and more packed in fluid phase, and there is virtually no difference in gel phase. In addition, while APL fluctuates throughout the simulation, the extent of the fluctuations (as seen from the standard deviation) is comparable for both systems. On the other hand, the simulations have shown much lower lateral diffusion of gel phase lipids in multilamellar systems consistent with experimental observations of tighter packing in MLV at lower temperatures. These observations combined serve as indications of higher rigidity and consequently lower deformability of MLVs. The lack of decisive results may be due to the nature of the computational setup: (i) the simulations encompass only the small section of the membrane where curvature will not be pronounced; (ii) the interactions between periodic images in ML system will be dampened by the twofold rotational symmetry in *xy* dimensions [76]; and (iii) all systems contain the same amount of Na^+^ bound to the membrane surface (Table S1). It is known that the increase in bending modulus may be greatly mediated by the presence of salt [86], and in the simulations, it was necessary to place the same number of Na^+^ ions to neutralize the membrane charge, despite recognizing that it changes the concentration (i.e., ionic strength) between the systems. When the observed phenomenon is placed in the context of the electromechanical properties of lipid bilayers, there is an instantaneous and significant change in curvature in LUV [25] associated with the local pH change [88] and/or bilayer thermal fluctuations [89] in anionic lipid bilayers. However, spatially inequivalent charge distributions originating from lateral interactions between charged lipids, which are additionally intertwined with the HB network of the interfacial water layer [18], ultimately promote the formation of curved features on the bilayer and/or induce significant changes in the bilayer elastic constants [24]. The immediate curvature change induces the variation in local pH value, the effect of which is especially frequent in inverted micelles and in liposomes negatively curved-inner leaflet [90], resulting not only with the occasional protonation of DPPS lipids that are, according to the more stretched signature of protonated COOH lipids (DSC curve in Figure 2d), distributed in a relatively heterogeneous immediate environment, but also with the defects in water-assisted HB network that might extend beyond of the first methylene groups in the hydrocarbon chain. Energetically unfavorable hydrophobic hydration could be amortized by the curvature fluctuations at the surface of LUVs, the effect of which is prevented in MLV due to the restrictions imposed by the adjacent bilayers and the confined water between them. Nevertheless, since lipid membranes are soft and easily deformable structures, even the fluctuations in thermal energy can assist in changing the curvature of the membrane surface [89,91]. In either case, it appears that LUV are more amenable to coupling the electrical and mechanical attributes of DPPS lipid bilayers than MLV. Ultimately, our interpretation of the obtained results does not conflict with the interpretation of the structural data published so far on LUVs made of POPS [92] and DOPS [93] lipids. More specifically, the natural asymmetry of POPS LUV resulting from the greater roughness of the inner leaflet compared to the outer one [92] can be interpreted in the context of structural properties as a change in the curvature of the surface, which due to the dynamics of the movement of lipid molecules changes over time, fluctuates. Additionally, compared to DOPS LUV, in which it was assumed that besides the change in curvature, the electrostatic interactions also play an important role, the latter might be significant in DPPS (+ 5% DPPG) as well; although Kučerka et al. argued that the contribution of electrostatic interactions to the intrinsic asymmetry of DOPS LUV should be significantly higher when the latter are suspended in a solution of lower ionic strength (that is, in pure water) [93], the positions and phase-dependent displacements of the bands originating from ν_(a)s_COO^−^ in LUV (Figure 3b,c) imply a non-negligible influence of electrostatics [25] and make a prominent difference between MLV and LUV. If the structural and dynamic features of inherent surface curvature fluctuations even above melting temperature stand as a decisive discriminating factor between electromechanical features of LUV and MLV [24], it is necessary to identify their molecular origin.

The signature of curvature change, which is presumably distinct in MLV and LUV, is to be elucidated from the FTIR spectra acquired when lipids are in L_β/c_ phase (35 °C) and in L_α_ phase (65 °C) [36]. Besides confirming the breaking of van der Waals interactions as a consequence of the L_β/c_ → L_α_ phase transition [68], the spectral region where the bands originating from ν_as_CH_2_ and symmetric ν_s_CH_2_ are observed looks quite predictable and equally so for MLV and LUV (Figure 3a).

Quite the opposite, signals originating from the glycerol backbone and the carboxyl group of DPPS reveal considerable differences. The signatures of non-HB and HB oscillators of the glycerol moiety, along with the signature of νC=O of COOH group, suggest qualitatively different hydration of glycerol backbone in MLV and LUV. First, a doublet with relatively sharp and well distinguished maxima at 35 °C in MLV (1742 cm^−1^ and 1716 cm^−1^) is inherent to the highly ordered structures, implying that DPPS lipids in MLV are found in a L_c_ phase. The significantly superimposed bands originating from the stretching of non- and HB C=O- oscillators at 35 °C in LUV (1741 cm^−1^) suggest lipid ordering in L_β_ phase. Upon melting (L_β/c_ → L_α_) in both MLV and LUV, DPPS lipids experience greater rotational mobility of acyl chains and high-amplitude reorientation fluctuations, thus producing broader band profiles in both MLV and LUV (65 °C) [68]. Moreover, the higher proportion of HB oscillators in LUVs than in MLVs at both temperatures (in both phases), which is further accompanied by a wing on the low-frequency side νC=O (Figure 3b, * in yellow rectangle), indicates greater participation of LUV glycerol backbone in HB network with interfacial water layer than the one of MLV [94]. The displacement of the ν_as_COO^−^ band of DPPS during the L_c_ → L_α_ transition in MLV (maximum at 1638 cm^−1^ at 35 °C appears at 1610 cm^−1^ at 65 °C) suggests a considerable increase in ionization degree [21,93,95] upon melting of MLV. As this finding does not apply for LUV (maximum at 1599 cm^−1^ at 35 °C appears at 1577 cm^−1^ at 65 °C), the melting of which starts from L_β_ phase, it might be that different kinds of ion pairs are formed among COO^−^ moiety of MLV/LUV and counterions present in solution [96,97,98] or that its hydration in MLV/LUV is qualitatively different which reflects on the bond length (see Section 5).

The difference in the packing pattern of lipid molecules in MLV and LUV is unraveled by the position of γCH_2_ band [99] (Figure 3c): at 35 °C, the lipids in MLV are arranged in a L_c_ phase (1472 cm^−1^), whereas in LUV they are in a L_β_ phase (1467 cm^−1^). Our finding matches with the results provided by Lewis et al. with one interesting and currently unexplained exception that our suspensions were not kept at low temperatures for some time (usually several hours) before starting the measurements [21]. In contrast to the displacement magnitude of ν_as_COO^−^ band, the ν_s_COO^−^ band of MLV seems to be insensitive to the phase change (from 1417 cm^−1^ at 35 °C to 1416 cm^−1^ at 65 °C), whereas its low-frequency displacement (from 1415 cm^−1^ at 35 °C to 1396 cm^−1^ at 65 °C) in LUV suggests by far greater transition dipole moment change of carboxyl moiety in LUV. The opposite displacement direction of δCOH in MLV (1492 cm^−1^ at 35 °C and 1494 cm^−1^ at 65 °C) and LUV (from 1505 cm^−1^ at 35 °C to 1492 cm^−1^ at 65 °C), supports the assumption that protonated DPPS molecules in MLV and LUV are included in different HB fashion pattern.

Finally, the region that contains ν_as_PO_2_^−^ and ν_(a)s_C−O bands reveals the differences in the hydration of the phosphate group in MLV and LUV on the basis of different shapes and positions of the band maxima: almost unshifted ν_as_PO_2_^−^ signature originated from non-HB and HB species upon melting in MLV (1236 cm^−1^/1236 cm^−1^ and 1222 cm^−1^/1221 cm^−1^ at 35 °C/65 °C) and merging of respective signatures in LUV and low-frequency displacement upon the L_β_ → L_α_ transition (the envelope with maxima at 1244 cm^−1^ and 1223 cm^−1^ at 35 °C transforms into the envelope with maximum at 1220 cm^−1^ at 65 °C, see Figure 3d). The signature ν_(a)s_C−O, not usually excessively used, on this occasion supports the assumption that the glycerol moiety of LUV is, presumably due to the formidable curvature fluctuations [36,67,94], exposed to the interfacial water layer to by far greater extent than in MLV according to the substantially larger low-frequency shifts of both ν_as_C−O and ν_s_C−O bands is LUV than in MLV (Figure 3d).

MD simulations were employed for the characterization of water at the membrane interface and evaluation of its interactions with particular lipid moieties. In both unilamellar and multilamellar systems, the lipids are fully hydrated and the water penetrates through to the glycerol backbone and through water density profiles, show slightly more water molecules surrounding UL system headgroups at 65 °C. This is in accordance with the slightly lower rigidity observed which facilitates water penetration. Consequently, the HB network between water and glycerol, PO_2_^−^, C=O and COO^−^ appears slightly more pronounced in UL simulations, particularly at 65 °C. Even though the described trends are consistent for all systems and analyses, it is still important to mention that the effects are small and fall within method error. The reasons for such small effects may again be the small size of the system which results in poorer statistics (several thousand molecules in simulation vs. several moles of molecules on the macroscopic level) and the unequal ionic strength as the result of membrane neutralization [75]. Furthermore, the limitations of classical MD do not allow for studying the effects of lipid protonation, nor the spontaneous membrane curvature that are significant contributors to HB formation. In the future, the model could be improved by substantially increasing the bilayer size and potentially employing coarse-grained MD to attempt to capture surface structural fluctuations at larger scales. In addition, reactive or ab initio MD might be considered for the exploration of protonation. However, the classical all-atom model is still valuable for providing insight into membrane structure and hydration. The HB analysis supports experimental findings of extensive HB networking between lipid headgroups and glycerol backbone with water. Regarding the observed changes of FTIR bands of COO^−^, the difference in hydrogen bonding from the simulations is not sufficiently strong enough to yield a definite conclusion. However, since no evidence of differing Na^+^ interactions was found, HB formation remains a more likely cause of the displacement of spectral bands.

Interestingly, water-water hydrogen bonding is significantly increased in UL systems even after normalizing to account for the difference in total number of water molecules. The cause of this discrepancy may be linked to the other effect noticed in simulations: pronounced water ordering in ML systems. Formation of HBs not only requires the appropriate vicinity of donor and acceptor groups, but also the correct angle, which may be harder to achieve in interbilayer spaces where the orientation of water is dictated by two membrane surfaces [100]. Though the headgroup-controlled water organization is the same within the first hydration shell (0.3 nm from the surface), in ML systems, water structure is perturbed throughout the interlamellar space [100,101]. In UL systems, there is enough space away from the membrane for the water to lose structuration and orient in near-random manner, promoting freer water-water interactions. Rigid organization of interbilayer water was also noted in the works of Ge and Freed [34], and here further confirmed by an almost two-fold reduction in water diffusion coefficients in ML systems compared to UL. The same effect on *D* was noted previously when comparing the movement of bulk and interfacial water [79,102], leading to the conclusion that water in UL simulations behaves predominantly as bulk water, while in ML simulations, the majority of water molecules behave as interfacial water. Thus, not only does the water in ML systems have higher level of ordering, but the lower mobility also results in prolonged contact with the lipid. The higher level of organization and stability in water networks spanning the entire interbilayer space are thus a likely contributing factor to higher membrane rigidity of MLVs.

## 6. Conclusions

This comprehensive thermoanalytical, spectroscopic and computational study highlighted the key differences between MLV and LUV prepared from the DPPS anionic lipid with 5% (*x*) of DPPG. The maximum of surface curvature fluctuations at MLV was recorded around 2 °C before the actual melting (UV/Vis) and, as far as we know, this work is the first in which this phenomenon was registered at all. The inability to unequivocally assign any discontinuous structural change other than melting in the LUV, due to the large uncertainty in UV/Vis measurements and on the high-temperature side of a DSC curve, can be interpreted as continuous manifestations of surface curvature fluctuations, i.e., time-varying structural features of an inherently unstable system. In addition to revealing a different packing pattern of DPPS lipids at 35 °C in MLV (L_c_) and LUV (L_β_), FTIR spectra showed that the glycerol backbone in LUV is more exposed to interfacial water, which was interpreted as a consequence of surface curvature fluctuations and is especially pronounced in the LUV fluid phase (65 °C). Along with supporting the findings observed from FTIR spectra, MD simulations of multilamellar and unilamellar membranes showed significant structuration of interbilayer water and reduction of its mobility, which may be contributing factors to the attenuation of membrane curvature in MLV. Extensive hydrogen bonding throughout lipid headgroups and glycerol backbone was also confirmed.

## Figures and Tables

**Table 1:** Melting temperatures (*T*_m_) of DPPS-constituted MLV and LUV obtained from DSC curves (by measuring both onsets (o; *T*_m,o_) and maxima (m; *T*_m,m_) of the main phase transition of deprotonated DPPS and by estimating the curve maximum (MLV)/interval (LUV) of protonated ones, respectively (*T*_m,H_) and those obtained from a double (MLV; *T*_m,1_ and *T*_m,2_) and single (LUV; *T*_m_) sigmoid fit of spectral projections of temperature-dependent UV/Vis spectra.

Suspension	*T*_m_^a^				
	DSC			UV/Vis (MCA)	
	*T*_m,o_	*T*_m,m_	*T*_m,H_	*T*_1_	*T*_2_
MLV	51.3 ± 0.1	52.4 ± 0.1	≈60	51.5 ± 0.3	53.3 ± 0.1
LUV	≈50–59		≈59–61	53.9 ± 0.8 ^b^	

^a^ In °C; ^b^ = *T*_m_.

**Table 2:** Area per lipid (APL) and membrane thickness of DPPS + 5% DPPG unilamellar (UL) and multilamellar (ML) systems at 35 and 65 °C determined computationally.

System	*T* ^a^	APL ^b^	Membrane Thickness ^c^
UL	35	0.458 ± 0.003	4.514 ± 0.006
	65	0.578 ± 0.013	4.057 ± 0.032
ML	35	0.462 ± 0.004	4.516 ± 0.007
	65	0.563 ± 0.013	4.194 ± 0.003

^a^ In °C; ^b^ In nm^2^; ^c^ In nm.

**Table 3:** Average number of hydrogen bonds formed between specific groups or molecules.

Hydrogen Bonds	UL 35 °C	UL 65 °C	ML 35 °C	ML 65 °C
Lipid-lipid total	217 ± 8	183 ± 11	219 ± 7	190 ± 10
Lipid-lipid per molecule	1.70 ± 0.06	1.43 ± 0.09	1.71 ± 0.06	1.48 ± 0.08
Water-water total	9604 ± 45	9061 ± 54	3630 ± 31	3371 ± 35
Water-water per molecule	1.50 ± 0.01	1.42 ± 0.01	1.31 ± 0.01	1.21 ± 0.01
Water-lipid	1315 ± 24	1430 ± 37	1296 ± 23	1395 ± 33
Water-PO_2_^−^	346 ± 12	398 ± 16	348 ± 12	390 ± 15
Water-C=O	130 ± 9	179 ± 11	123 ± 9	175 ± 10
Water-COO^−^	552 ± 13	537 ± 17	544 ± 13	523 ± 16
Water-glycerol	147 ± 9	202 ± 11	139 ± 10	197 ± 11

**Table 4:** Diffusion coefficients *D* for water and lateral diffusion coefficients *D_xy_* in unilamellar and multilamellar systems at different temperatures.

System	*T* ^a^	*D* (Water) ^b^	*D_xy_* (Lipid) ^c^
UL	35	3.12 ± 0.04 × 10^−5^	0.91 ± 0.05 × 10^−7^
	65	4.16 ± 0.06 × 10^−5^	4.24 ± 0.14 × 10^−7^
ML	35	1.83 ± 0.09 × 10^−5^	0.25 ± 0.05 × 10^−7^
	65	2.30 ± 0.03 × 10^−5^	3.94 ± 0.33 × 10^−7^

^a^ In °C; ^b^ In cm^2^ s^−1^; ^c^ In nm.

## Data Availability

Not applicable.

## Supplementary Materials

The following supporting information can be downloaded at: https://www.mdpi.com/article/10.3390/membranes13010083/s1, Figure S1: DLS data on: (a) MLV and (b) LUV prepared from DPPS lipids; Figure S2: Confocal microscope images of: (a) MLV and (b) LUV of DPPS (with 5% DPPG) measured in transmissive mode (details on the measurements are presented in the main text, Section 2.2 Dynamic light scattering (DLS) and confocal microscopy: Measurements and data analysis); Figure S3: DSC curve of MLV in the temperature range 25–65 °C (the phase transitions temperatures are listed and highlighted with dashed lines); Figure S4: DSC curve of LUV (the phase transitions temperatures are listed and highlighted with dashed lines): (a) after 5 h of standing, (b) after 24 h of standing; Figure S5: DSC curves of MLV collected in duplicates (first measurement: violet curve; second measurement: pink curve: (a) first heating run; (b) first cooling run; (c) second heating run; (d) second cooling run. Phase transitions maxima are highlighted with dashed lines and associated temperatures are written on graphs; Figure S6: DSC curves of LUV collected in duplicates (first measurement: olive curve; second measurement: green curve: (a) first heating run; (b) first cooling run; (c) second heating run; (d) second cooling run. Phase transitions maxima are highlighted with dashed lines and associated temperatures are written on graphs; Figure S7: Snapshots of the final frames of MD production runs for DPPS + 5% DPPG bilayers for: (a) unilamellar system at 35 °C, (b) unilamellar system at 65 °C, (c) multilamellar system at 35 °C and (d) multilamellar system at 65 °C. Two periodic images of each system are partially displayed (transparent); Figure S8: Deuterium order parameters (-SCD) for carbon atoms in DPPS and DPPG acyl chains: (a) chain 1 (upper chain on each structure from Figure 1); (b) chain 2 (bottom chain on each structure from Figure 1); Figure S9: Mass density profiles of DPPS and DPPG headgroups, water and Na^+^ along *z*-axis: (a) unilamellar system at 35 °C, (b) unilamellar system at 65 °C, (c) multilamellar system at 35 °C and (d) multilamellar system at 65 °C. The values for DPPG headgroups have been increased 10-fold to improve visibility on the graph; Figure S10: Radial distribution functions of water oxygen with membrane lipids as reference; Figure S11: Cosine of the angle between water dipole vector and *z*-axis, as a function of *z*-axis length: (a) unilamellar system at 35 °C, (b) unilamellar system at 65 °C, (c) multilamellar system at 35 °C and (d) multilamellar system at 65 °C; Table S1: The average number of Na^+^ ions in contact with DPPS COO^−^ groups.
