# Supplementary material for: Lamellarity-Driven Differences in Surface Structural Features of DPPS Lipids: Spectroscopic, Calorimetric and Computational Study
Source: Membranes (Basel). 2023 Jan 9;13(1):83. doi: 10.3390/membranes13010083 (PMC9865892; doi:10.3390/membranes13010083)
Supplement: Supplementary file 1 [file membranes-13-00083-s001.zip › membranes-2081980-supplementary.pdf]

Lamellarity-driven differences in surface structural features of DPPS lipids: spectroscopic, calorimetric and computational study

Lea Pašalić, Barbara Pem, Danijela Bakarić*

Division for Organic Chemistry and Biochemistry, Ruđer Bošković Institute, Bijenička 54, 10000 Zagreb, Croatia

*Corresponding author. Tel.: +385 1 4571 382; E-mail: danijela.bakaric@irb.hr (Danijela Bakarić)

Supporting Information

S1. DLS data on MLV and LUV p2

S2. Confocal microscopic imaging of MLV and LUV p3

S3. Auxiliary DSC data of MLV and LUV p4

S4. Auxiliary MD data p9

References p13

S1. DLS data on MLV and LUV

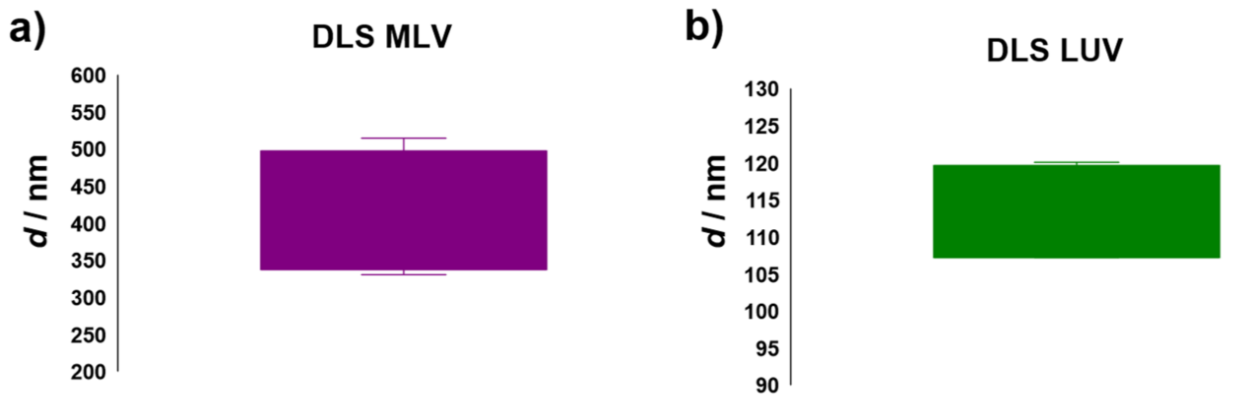

Figure S1: DLS data on: a) MLV and b) LUV prepared from DPPS lipids.

We would also like to point out that we measured DLS of LUV before each measurement (after each extrusion process). More or less we got the same value regarding their average diameter (*d* / nm).

S2. Confocal microscopic imaging of MLV and LUV

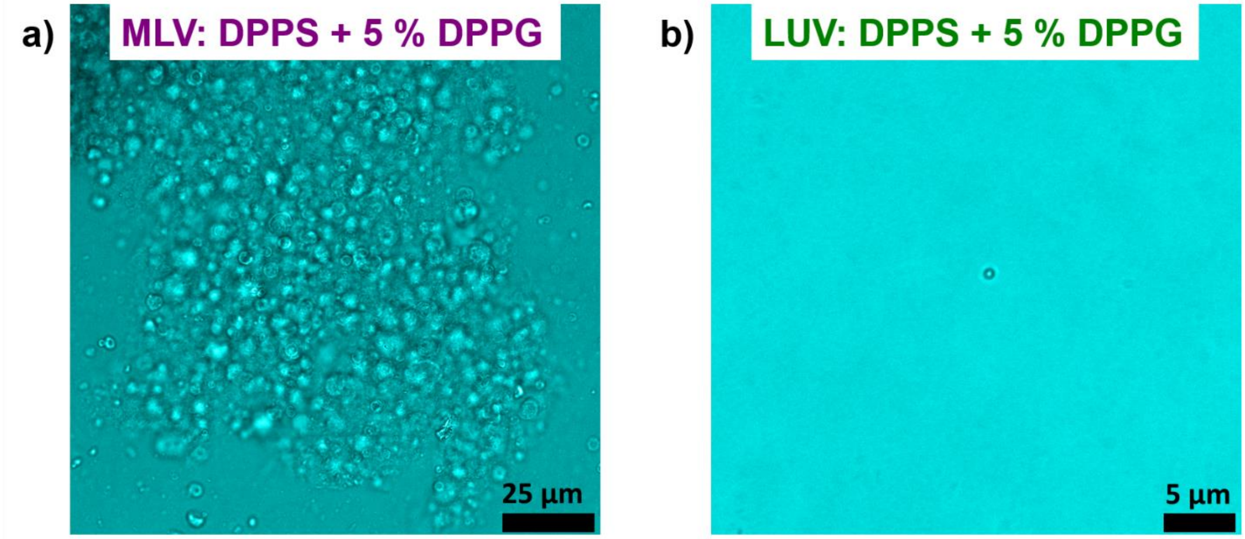

Figure S2: Confocal microscope images of: a) MLV and b) LUV of DPPS (with 5 % DPPG) measured in transmissive mode (details on the measurements are presented in the main text, subsection 2.2 Dynamic light scattering (DLS) and confocal microscopy: Measurements and data analysis).

S3. Auxiliary DSC data of MLV and LUV

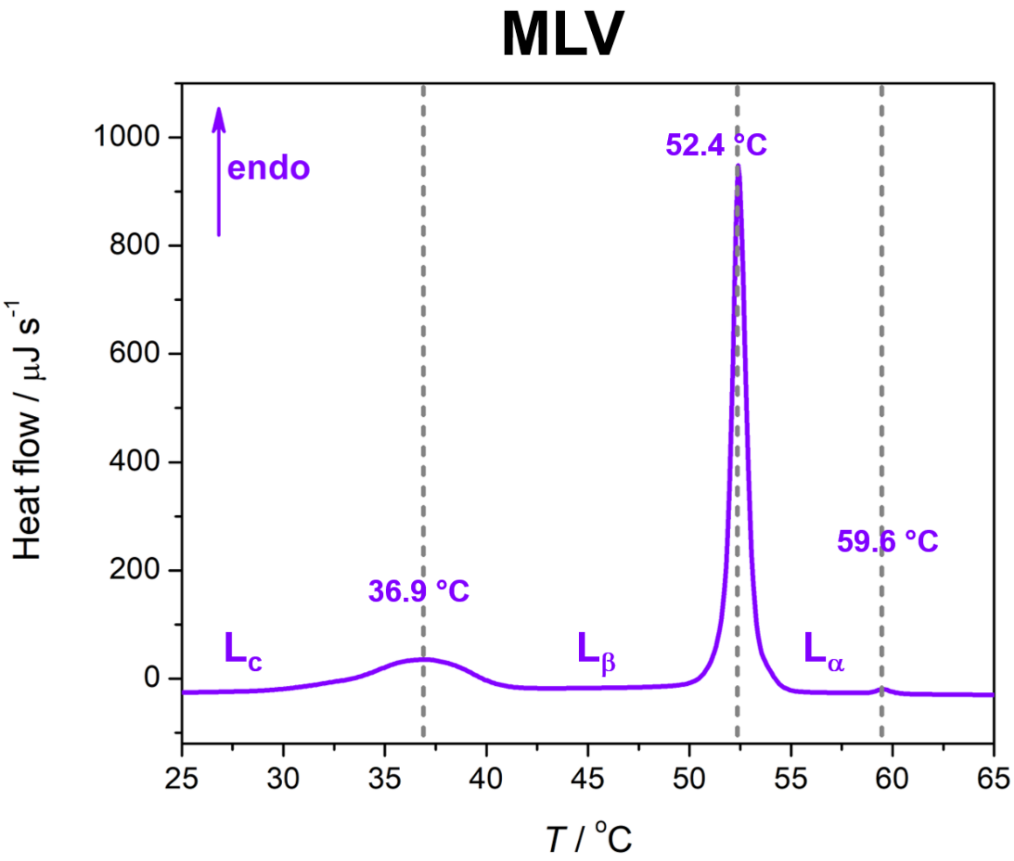

Figure S3: DSC curve of MLV in the temperature range 25-65 °C (the phase transitions temperatures are listed and highlighted with dashed lines).

The phase transitions $L_c \rightarrow L_\beta$ and $L_\beta \rightarrow L_\alpha$ of deprotonated DPPS lipids occur at 36.9 °C and 52.4 °C, respectively, whereas $L_\beta \rightarrow L_\alpha$ phase transition of protonated ones appears at 59.6 °C [1–3].

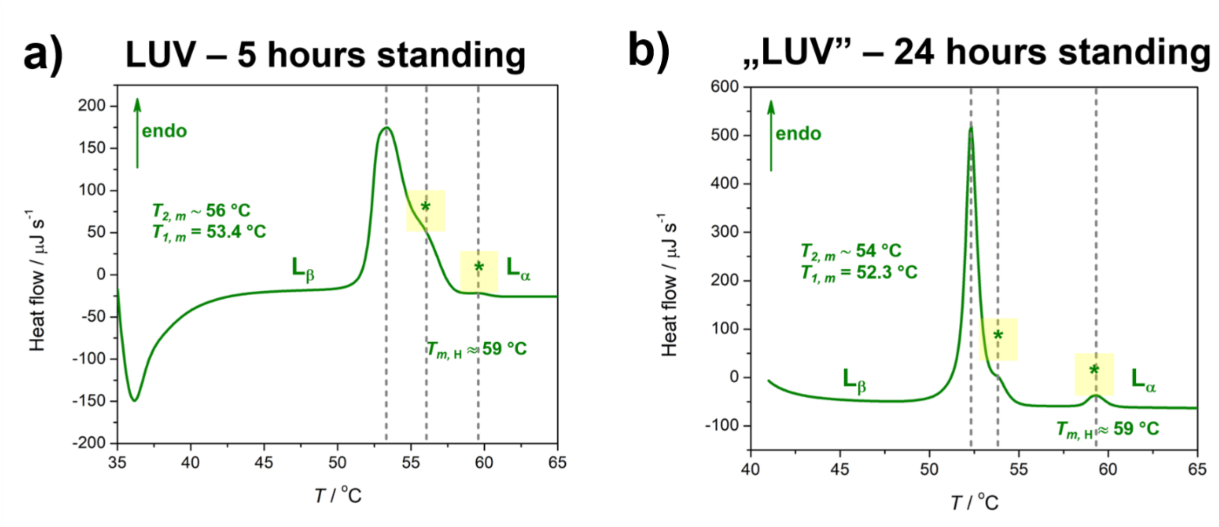

Figure S4: DSC curve of LUV (the phase transitions temperatures are listed and highlighted with dashed lines): a) after 5 hours of standing, b) after 24 hours of standing.

DSC curve in Fig. S4 reported the thermotropic behavior of LUV (and „LUV") constructed from DPPS (+ 5% DPPG) lipids after 5 hours (a) and 24 hours (b) of refrigeration. Since the curve on Fig. 4a) qualitatively resembles the average DSC curve of LUV examined immediately after extrusion (see Fig. 2d), we conclude that the LUV are stable/do not lamellate within 6 hours, i.e. the time required to complete the DSC measurement. After 24 hours, however, LUV obviously undergo lamellarization and, accordingly, we named them „LUV" (compare with Fig. S3).

The phase transition $L_\beta \rightarrow L_\alpha$ of deprotonated DPPS lipids in LUV/"LUV" starts at about 50 °C and ends at about 59 °C. The melting appears to be the superposition of two distinct events: the one at 53.4 °C/52.3 °C (presumably originated from the curvature change) and another at 56 °C/54 °C (labeled with * in a yellow rectangle, presumably due to the breaking of van der Waals forces). The melting of protonated DPPS lipids is detected at about 59 °C/59 °C.

Unlike in MLV, there are no traces of lipids arranged in L_c phase (compare with the Fig. S3).

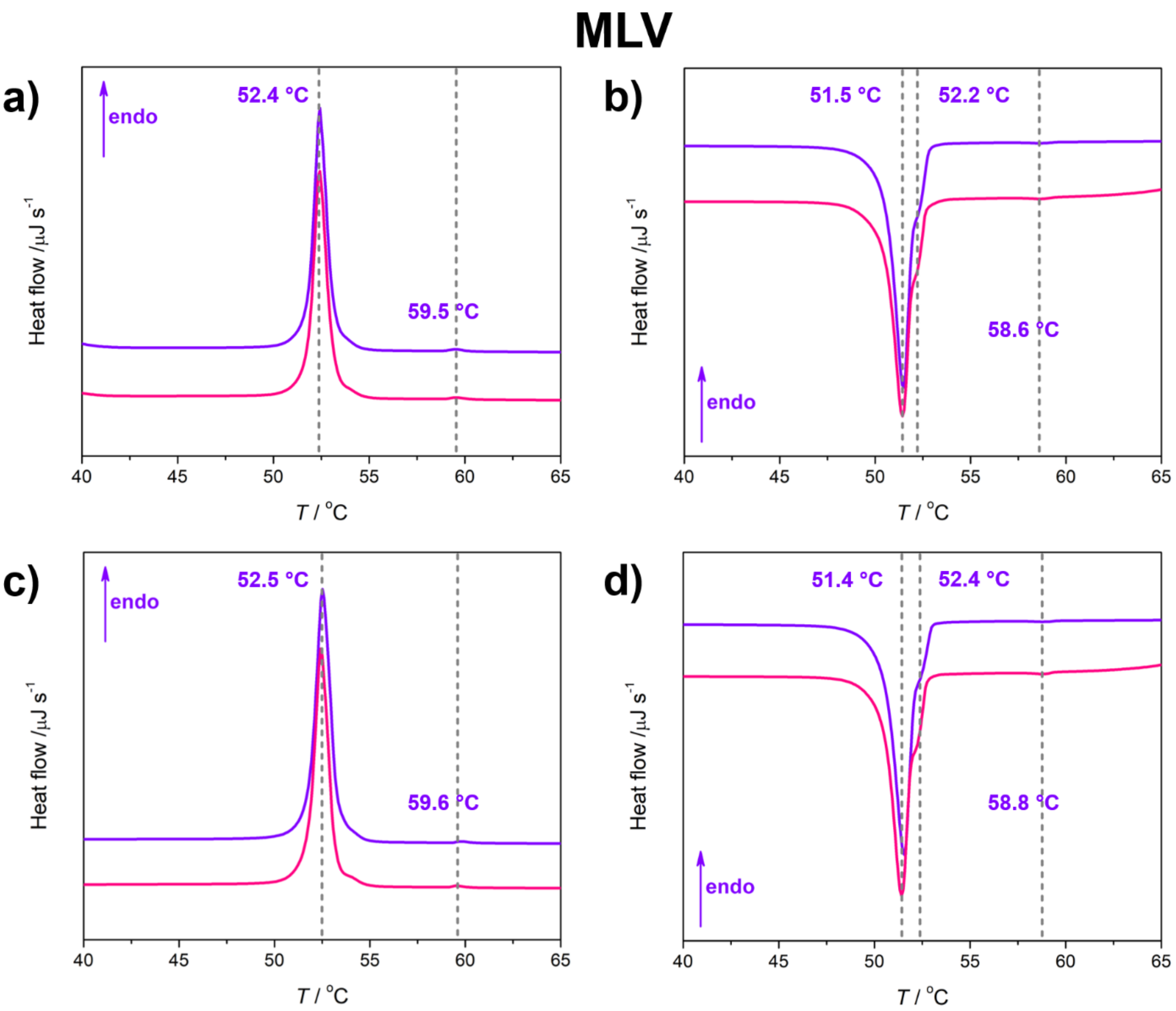

Figure S5: DSC curves of MLV collected in duplicates (first measurement: violet curve; second measurement: pink curve: a) first heating run; b) first cooling run; c) second heating run; d) second cooling run. Phase transitions maxima are highlighted with dashed lines and associated temperatures are written on graphs.

The duplicates of DSC curves of MLV during the first and second heating and cooling runs very much resemble each other (the values are listed on corresponding graph), suggesting remarkable reproducibility and MLV stability. Beside melting ($L_\beta \leftrightarrow L_\alpha$) of deprotonated DPPS ($T_m$ = 52.4 °C (a), 51.5 °C (b), 52.5 °C (c) and 51.4 °C (d)) and protonated one ($T_{m,\,H}$ = 59.5 °C (a), 58.6 °C (b), 59.6 °C (c) and 58.8 °C (d)) in the cooling runs there are traces of, presumably, unharmonicity in ordering of DPPS lipids (52.2 °C (b) and 52.4 °C (d)).

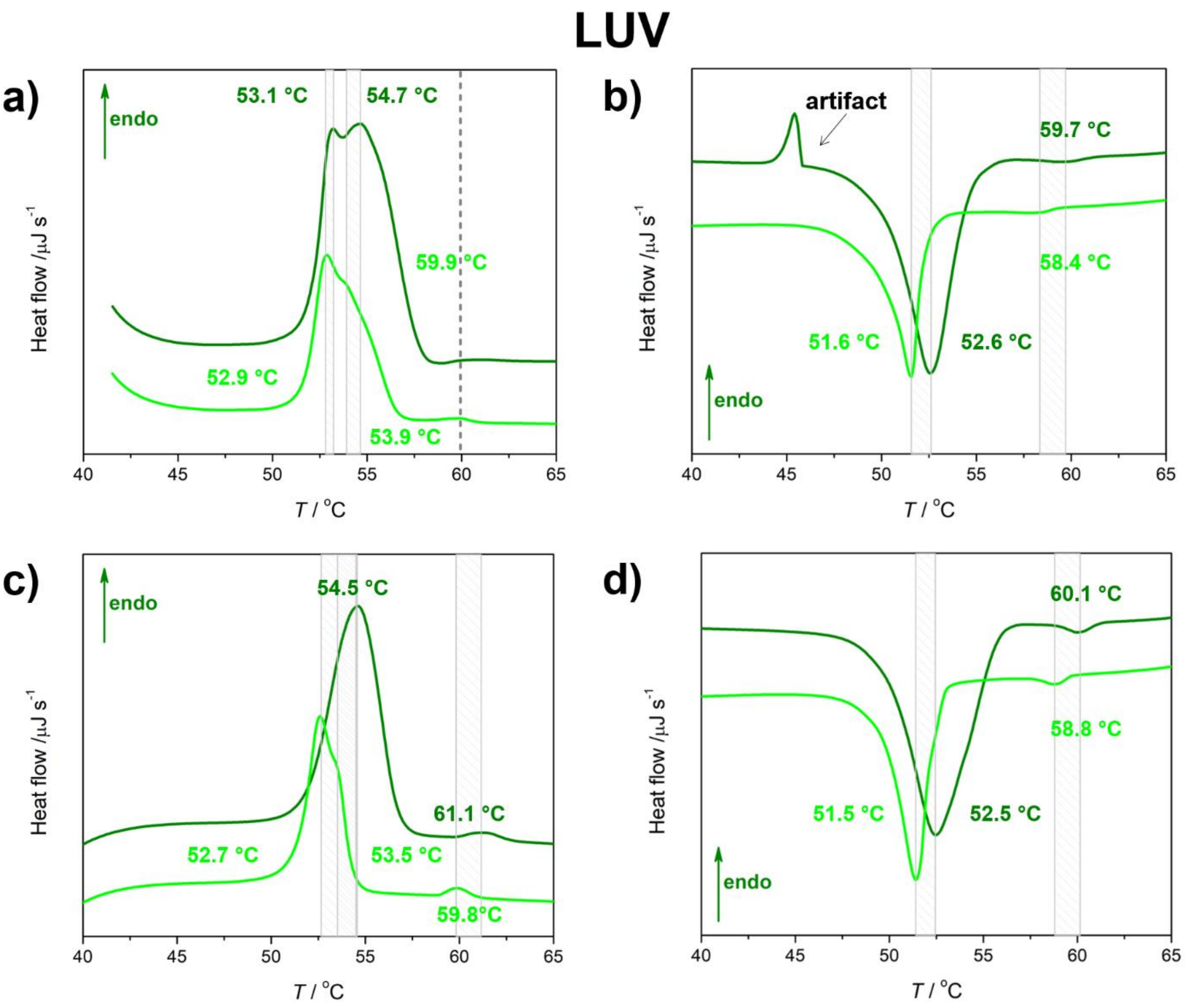

Figure S6: DSC curves of LUV collected in duplicates (first measurement: olive curve; second measurement: green curve: a) first heating run; b) first cooling run; c) second heating run; d) second cooling run. Phase transitions maxima are highlighted with dashed lines and associated temperatures are written on graphs.

Unlike in MLV, the duplicates of DSC curves of LUV display certain qualitative differences. First, the heating runs do not look exactly the same, although general curve broadness at the appearance of two possible separate transitions (mean curvature change and the breaking of van der Waals interactions) is suggested, along with the melting of protonated DPPS lipids: a) 52.9 °C/53.1 °C, 53.9 °C/54.7 °C and 59.9 °C/– °C during the first/second measurement in the first heating run (melting of protonated DPPS in the first measurement of the first heating run is barely visible); c) 52.7 °C/54.5 °C, 53.5 °C/54.5 °C and 59.8 °C/61.1 °C during the first/second measurement in the

second heating run (the second measurement resulted with a broad curve with maximum at 54.5 °C); b) 58.4 °C/59.7 °C and 51.6 °C/52.6 °C during the first/second measurement in the first cooling run (an artifact present is highlighted); b) 58.8 °C/60.1 °C and 51.5 °C/52.5 °C during the first/second measurement in the second cooling run. The variability in these separatelly presented measurements reflects strong instability of LUV.

S4. Auxiliary MD data

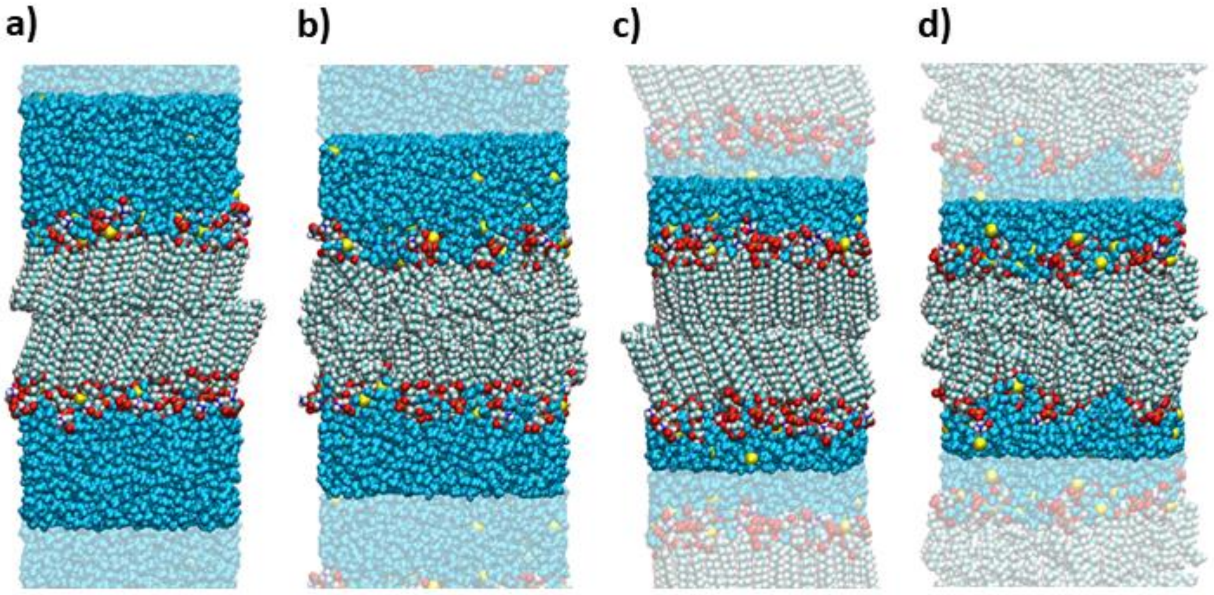

Figure S7: Snapshots of the final frames of MD production runs for DPPS + 5% DPPG bilayers for a) unilamellar system at 35 °C, b) unilamellar system at 65 °C, c) multilamellar system at 35 °C and d) multilamellar system at 65 °C. Two periodic images of each system are partially displayed (transparent).

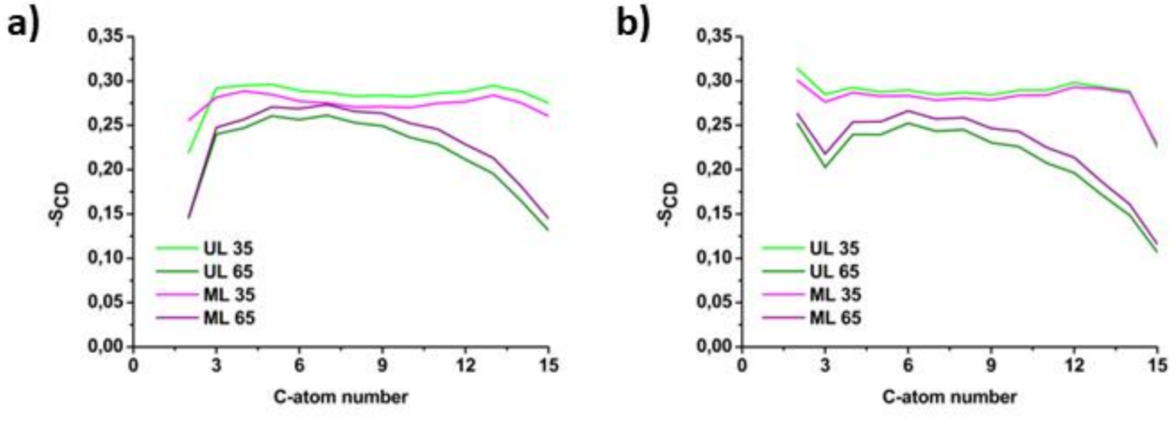

Figure S8: Deuterium order parameters ($-S_{CD}$) for carbon atoms in DPPS and DPPG acyl chains: a) chain 1 (upper chain on each structure from Fig. 1); b) chain 2 (bottom chain on each structure from Fig. 1).

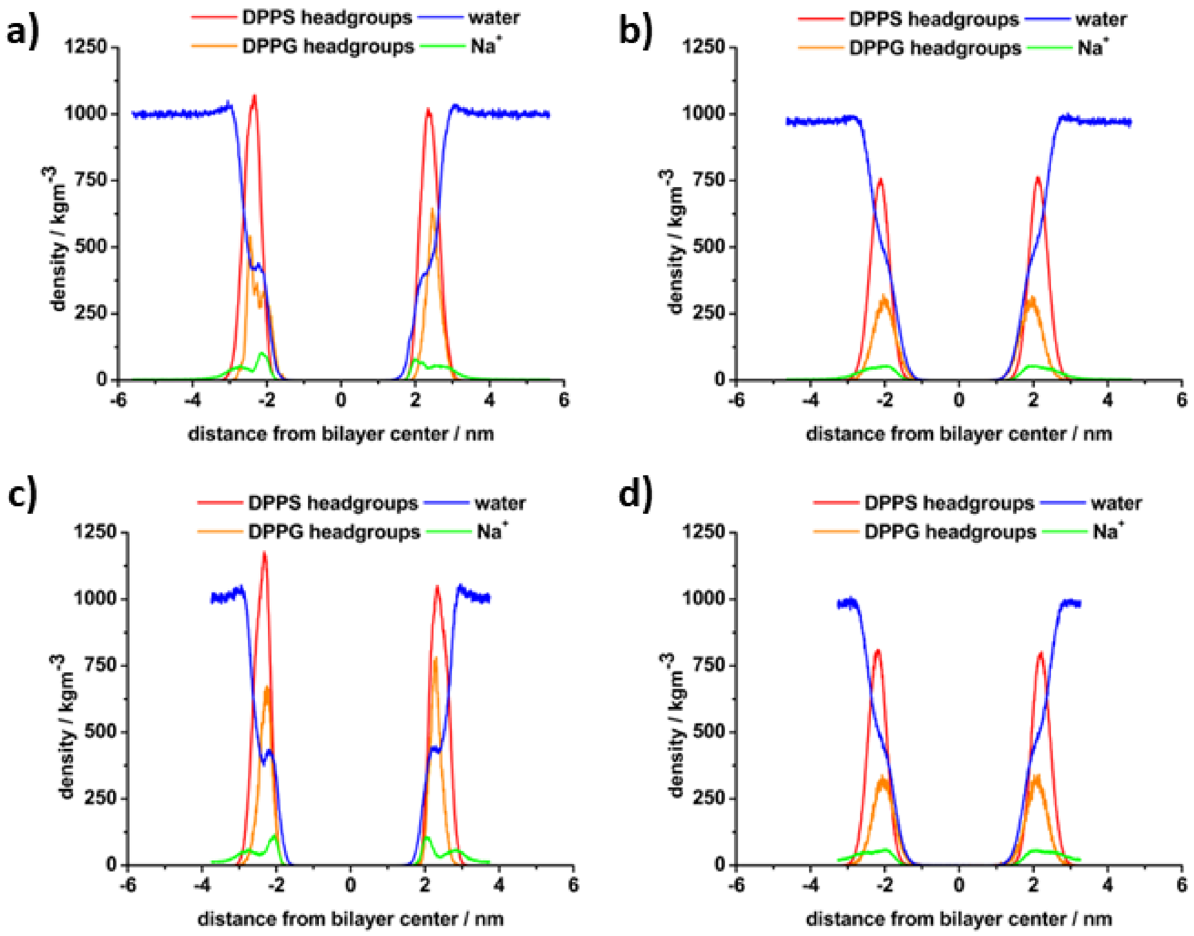

Figure S9: Mass density profiles of DPPS and DPPG headgroups, water and Na^+ along *z*-axis: a) unilamellar system at 35 °C, b) unilamellar system at 65 °C, c) multilamellar system at 35 °C and d) multilamellar system at 65 °C. The values for DPPG headgroups have been increased 10-fold to improve visibility on the graph.

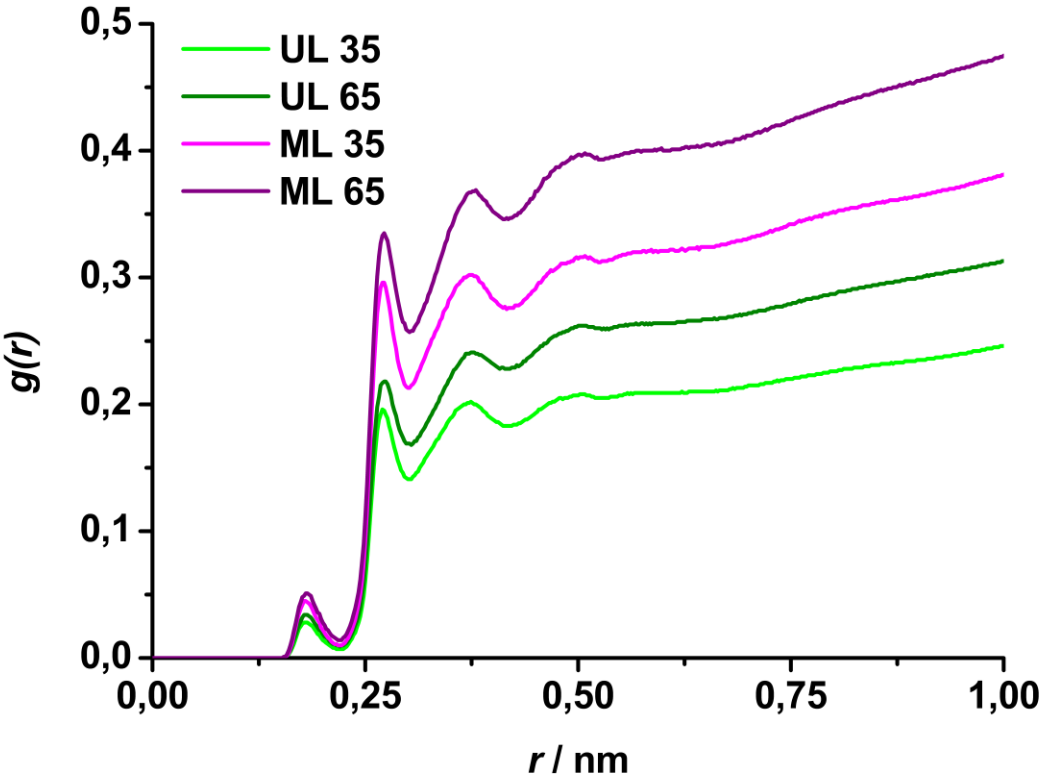

Figure S10: Radial distribution functions of water oxygen with membrane lipids as reference.

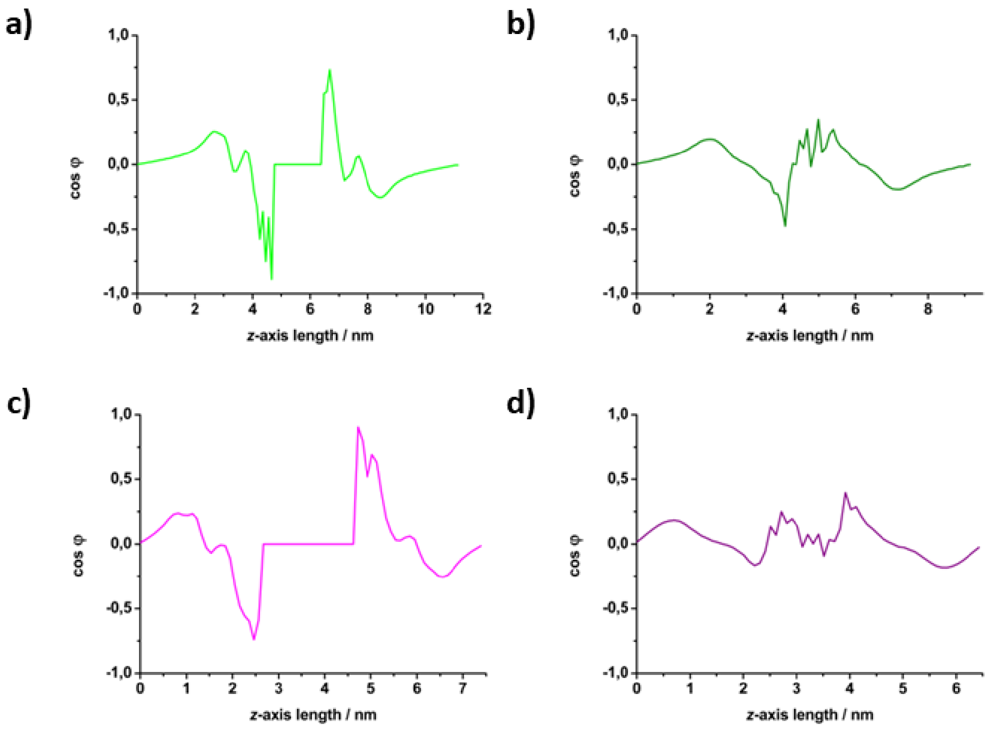

Figure S11: Cosine of the angle between water dipole vector and *z*-axis, as a function of *z*-axis length: a) unilamellar system at 35 °C, b) unilamellar system at 65 °C, c) multilamellar system at 35 °C and d) multilamellar system at 65 °C.

Table S1. The average number of Na^+ ions in contact with DPPS COO^- groups.

No. of ions	UL 35 °C	UL 65 °C	ML 35 °C	ML 65 °C
Na^+ around PO_4^-	47.7 ± 3.3	41.1 ± 4.6	46.0 ± 3.7	43.8 ± 4.7
Na^+ around C=O	40.1 ± 2.9	29.4 ± 3.8	44.4 ± 3.0	30.4 ± 3.7
Na^+ around COO^-	19.2 ± 3.6	23.2 ± 4.1	18.7 ± 3.4	25.2 ± 4.3

The Na^+-group contact was defined as the distance of 0.4 nm or less between the select two moieties. 0.4 nm was chosen as the edge of the first interaction shell (first minima from Na^+-PO_4^-, Na^+-C=O and Na^+-COO^- RDFs, data not shown).

References:

1. Lewis, R.N.A.H.; McElhaney, R.N. Calorimetric and Spectroscopic Studies of the Thermotropic Phase Behavior of Lipid Bilayer Model Membranes Composed of a Homologous Series of Linear Saturated Phosphatidylserines. *Biophys. J.* **2000**, *79*, 2043–2055, doi:10.1016/S0006-3495(00)76452-6.

2. Mitomo, H.; Kobayashi, S.; Iwayanagi, S.; Taktzawa, T.; Hayashi, K. Structural Changes in Phosphatidylserine Salts by Hydration and Addition of Calcium Ion. *J. Phys. Soc. Japan* **1993**, *62*, 2174–2179, doi:10.1143/JPSJ.62.2174.

3. Cevc, G.; Watts, A.; Marsh, D. Titration of the Phase Transition of Phosphatidylserine Bilayer Membranes. Effects of pH, Surface Electrostatics, Ion Binding, and Head-Group Hydration. *Biochemistry* **1981**, *20*, 4955–4965.
